# Cloxacillin plus fosfomycin versus cloxacillin alone for methicillin-susceptible *Staphylococcus* *aureus* bacteremia: a randomized trial

**DOI:** 10.1038/s41591-023-02569-0

**Published:** 2023-10-02

**Authors:** Sara Grillo, Miquel Pujol, Josep M. Miró, Joaquín López-Contreras, Gorane Euba, Oriol Gasch, Lucia Boix-Palop, Maria José Garcia-País, Maria Teresa Pérez-Rodríguez, Silvia Gomez-Zorrilla, Isabel Oriol, Luis Eduardo López-Cortés, Maria Luisa Pedro-Botet, Rafael San-Juan, José María Aguado, Francesca Gioia, Simona Iftimie, Laura Morata, Alfredo Jover-Sáenz, Graciano García-Pardo, Belén Loeches, Álvaro Izquierdo-Cárdenas, Ane Josune Goikoetxea, Aina Gomila-Grange, Beatriz Dietl, Damaris Berbel, Sebastian Videla, Pilar Hereu, Ariadna Padullés, Natalia Pallarès, Cristian Tebé, Guillermo Cuervo, Jordi Carratalà, SAFO study group, Maria Alba Rivera, Malen Aguirregabiria, Regino Rodríguez-Álvarez, María José Blanco-Vidal, Marina Alguacil-Guillen, Mariona Xercavins, Virginia Pomar, Ana Siverio-Parés, Marina de Cueto, Elisa Moreno-Mellado, Adrián Sousa, Francisco José Vasallo-Vidal, Beatriz Borjabad, Ana Coloma-Conde, Raquel Clivillé-Abad, Sabina Ximena González-di Lauro, Jose Tiago-Silva, Maria Angeles Orellana, Mario Ruíz-Bastián, Pilar Vizcarra, Carles Garcia, Frédéric Ballester, María Fernanda Ramírez-Hidalgo, Alba Bellés-Bellés, Yolanda Meije, Alba Ribera, Jaume LLaberia, María Ángeles Domínguez, Raul Francisco Rigo-Bonnin, Gertrudis Horna, Dominica Mediavilla, Mireia Sanllorente, Ester Picó-Plana, Alex Soriano, Cristina Pitart, Ana Maria Sanchez-Diaz

**Affiliations:** 1 Department of Infectious Diseases, Hospital Universitari de Bellvitge, Barcelona, Spain; 2 Centro de Investigación Biomédica en Red de Enfermedades Infecciosas (CIBERINFEC), Instituto de Salud Carlos III, Madrid, Spain; 3 Bellvitge Institute for Biomedical Research (IDIBELL), Barcelona, Spain; 4 University of Barcelona, Barcelona, Spain; 5 Department of Infectious Diseases, Hospital Clinic de Barcelona, Barcelona, Spain; 6 Institut d’Investigacions Biomèdiques August Pi i Sunyer (IDIBAPS), Barcelona, Spain; 7 Department of Infectious Diseases, Hospital de la Santa Creu i Sant Pau, Barcelona, Spain; 8 Institut d’Investigació Biomèdica Sant Pau, Barcelona, Spain; 9 Department of Infectious Diseases, Hospital Universitario Cruces, Barakaldo, Spain; 10 Biocruces Bizkaia Health Research Institute, Barakaldo, Spain; 11 Department of Infectious Diseases, Hospital Universitari Parc Taulí, Sabadell, Spain; 12 Department of Medicine, Universitat Autònoma de Barcelona, Barcelona, Spain; 13 Institut d’Investigació i Innovació Parc Taulí (I3PT), Sabadell, Spain; 14 Department of Infectious Diseases, Hospital Universitari Mútua Terrassa, Terrassa, Spain; 15 Department of Internal Medicine, Hospital Universitario Lucus Augusti, Lugo, Spain; 16 Instituto de Investigación Sanitaria de Santiago de Compostela, Santiago de Compostela, Spain; 17 Infectious Diseases Unit, Internal Medicine Department, Complexo Hospitalario Universitario de Vigo, Vigo, Spain; 18 Galicia Sur Health Research Institute, Vigo, Spain; 19 Institut Hospital del Mar d’Investigacions Mèdiques (IMIM), Universitat Pompeu Fabra (UPF), Barcelona, Spain; 20 Infectious Diseases Service, Hospital del Mar, Infectious Pathology and Antimicrobial Research Group (IPAR), Barcelona, Spain; 21 Department of Internal Medicine, Hospital de Sant Joan Despi Moises Broggi, Sant Joan Despi, Spain; 22 Infectious Diseases and Microbiology Clinical Unit, University Hospital Virgen Macarena, Seville, Spain; 23 Department of Medicine, School of Medicine, University of Sevilla, Biomedicine Institute of Seville (IBiS)/CSIC, Seville, Spain; 24 Hospital Universitari Germans Trias i Pujol, Badalona, Spain; 25 Fundació Institut d’Investigació en Ciències de la Salut Germans Trias i Pujol, Badalona, Spain; 26 Unit of Infectious Diseases, Hospital Universitario “12 de Octubre”, Madrid, Spain; 27 Department of Medicine, School of Medicine, Universidad Complutense, Madrid, Spain; 28 Instituto de Investigación Sanitaria Hospital “12 de Octubre” (imas12), Madrid, Spain; 29 Department of Infectious Diseases, Hospital Universitario Ramón y Cajal, Madrid, Spain; 30 Instituto Ramon y Cajal de Investigacion Sanitaria, Madrid, Spain; 31 Institut d’Investigació Sanitària Pere Virgili, Department of Medicine and Surgery, Universitat Rovira i Virgili, Reus, Spain; 32 Department of Internal Medicine, Hospital Universitari de Sant Joan, Reus, Spain; 33 Unidad Territorial Infección Nosocomial y Política Antibiòtica (UTIN), Hospital Universitari Arnau de Vilanova, Lleida, Spain; 34 IISPV, Universitat Rovira i Virgili, Tarragona, Spain; 35 Grup de control de la Infecció, Hospital Universitari de Tarragona Joan XXIII, Tarragona, Spain; 36 Department of Infectious Diseases, Hospital Universitario La Paz, Madrid, Spain; 37 Department of Microbiology and Parasitology, Hospital Universitari de Bellvitge (IDIBELL), Barcelona, Spain; 38 Centro de Investigación Biomédica en Red de Enfermedades Respiratorias (CIBERES), Instituto de Salud Carlos III, Madrid, Spain; 39 Spanish Clinical Research Network (SCReN), Hospital Universitari de Bellvitge (IDIBELL), Barcelona, Spain; 40 Department of Clinical Pharmacology, Clinical Research and Clinical Trials Unit, Barcelona, Spain; 41 Department of Pharmacy, Hospital Universitari de Bellvitge (IDIBELL), Barcelona, Spain; 42 Biostatistics Unit, IDIBELL, Barcelona, Spain; 43 Microbiology Department, Hospital de la Santa Creu i Sant Pau, Barcelona, Spain; 44 Microbiology Department, Hospital Universitario Cruces, Barakaldo, Spain; 45 Institut d’Investigació iInnovació Parc Taulí (I3PT), Sabadell, Spain; 46 Microbiology Department, Hospital Universitari Parc Taulí, Sabadell, Spain; 47 Microbiology Department, CatLab, Terrassa, Spain; 48 Laboratori de Referència de Catalunya, Universitat Pompeu Fabra (UPF), Barcelona, Spain; 49 Microbiology Department, Universitat Pompeu Fabra (UPF), Barcelona, Spain; 50 Complexo Hospitalario Universitario de Vigo, Galicia Sur Health Research Institute, Vigo, Spain; 51 Microbiology Department, Galicia Sur Health Research Institute, Vigo, Spain; 52 Microbiology Department, Consorci del Laboratori Intercomarcal de l’Alt Penedès, l’Anoia i el Garraf, Hospital Sant Joan Despí Moisès Broggi, Sant Joan Despi, Spain; 53 Hospital Universitario “12 de Octubre”, Instituto de Investigación Sanitaria Hospital “12 de Octubre” (imas12), Madrid, Spain; 54 Microbiology Department, Instituto de Investigación Sanitaria Hospital “12 de Octubre” (imas12), Madrid, Spain; 55 Microbiology Department, Hospital Universitario La Paz, Madrid, Spain; 56 Microbiology Department, Hospital Universitari de Sant Joan, Reus, Spain; 57 Microbiology Department, Hospital Universitari Arnau de Vilanova, Lleida, Spain; 58 Infectious Disease Unit, Internal Medicine Department, Hospital de Barcelona, Societat Cooperativa d’Instal·lacions Assistencials Sanitàries (SCIAS), Barcelona, Spain; 59 Microbiology Unit, Clinical Laboratory Department, Hospital de Barcelona, Societat Cooperativa d’Instal·lacions Assistencials Sanitàries (SCIAS), Barcelona, Spain; 60 Clinical Laboratory, Hospital Universitari de Bellvitge (IDIBELL), Barcelona, Spain; 61 Microbiology Department, Hospital Universitari de Tarragona Joan XXIII, Tarragona, Spain; 62 Institute of Global Health of Barcelona, Barcelona, Spain; 63 Microbiology Department, Hospital Clínic i Provincial, Barcelona, Spain; 64 Microbiology Department, Hospital Universitario Ramón y Cajal, Madrid, Spain

**Keywords:** Antimicrobial therapy, Randomized controlled trials

## Abstract

Treatment failure occurs in about 25% of patients with methicillin-susceptible *Staphylococcus* *aureus* (MSSA) bacteremia. We assessed whether cloxacillin plus fosfomycin achieves better treatment success than cloxacillin alone in hospitalized adults with MSSA bacteremia. We conducted a multicenter, open-label, phase III–IV superiority randomized clinical trial. We randomly assigned patients (1:1) to receive 2 g of intravenous cloxacillin alone every 4 h or with 3 g of intravenous fosfomycin every 6 h for the initial 7 days. The primary endpoint was treatment success at day 7, a composite endpoint with the following criteria: patient alive, stable or with improved quick Sequential Organ Failure Assessment score, afebrile and with negative blood cultures for MSSA, adjudicated by an independent committee blinded to treatment allocation. We randomized 215 patients, of whom 105 received cloxacillin plus fosfomycin and 110 received cloxacillin alone. We analyzed the primary endpoint with the intention-to-treat approach in 214 patients who received at least 1 day of treatment. Treatment success at day 7 after randomization was achieved in 83 (79.8%) of 104 patients receiving combination treatment versus 82 (74.5%) of 110 patients receiving monotherapy (risk difference 5.3%; 95% confidence interval (CI), –5.95–16.48). Secondary endpoints, including mortality and adverse events, were similar in the two groups except for persistent bacteremia at day 3, which was less common in the combination arm. In a prespecified interim analysis, the independent committee recommended stopping recruitment for futility prior to meeting the planned randomization of 366 patients. Cloxacillin plus fosfomycin did not achieve better treatment success at day 7 of therapy than cloxacillin alone in MSSA bacteremia. Further trials should consider the intrinsic heterogeneity of the infection by using a more personalized approach. ClinicalTrials.gov registration: NCT03959345.

## Abstract (web summary)

New treatments are essential for methicillin-susceptible *Staphylococcus* *aureus* bacteremia, but progress is slow. In this phase III–IV trial, cloxacillin plus fosfomycin failed to show superiority over cloxacillin alone, underscoring the challenges to improving patient outcomes.

## Main

*Staphylococcus* *aureus* is a major cause of life-threatening community-acquired and healthcare-associated bacteremia. The incidence of *S.* *aureus* bacteremia is increasing, ranging from 10 to 30 per 100,000 person-years^1^. The mortality rate associated with *S.* *aureus* bacteremia remains particularly high, ranging from 20% to 33% at 90 days, and is a matter of great concern^2,3^. This high mortality rate may be attributed to various factors, including increasing age and a higher frequency of comorbid conditions^4^. A poor prognosis of *S.* *aureus* bacteremia has also been linked to high-risk sources of infection, particularly endocarditis, pneumonia and cases of unknown origin^5^. Furthermore, persistent and complicated *S.* *aureus* bacteremia presents a major mortality risk,^6,7^ with each day of persistent bacteremia associated with a 16% increase in risk of death^6^.

Anti-staphylococcal beta-lactam monotherapy is currently considered the standard of care for the treatment of methicillin-susceptible *S.* *aureus* (MSSA) bacteremia^8^. However, treatment failure and mortality rates in MSSA bacteremia remain unacceptably high^9^. Consequently, there is growing interest in identifying new therapeutic regimens capable of reducing treatment failure and improving outcomes obtained with cloxacillin monotherapy. Experimental and clinical studies have found several antibiotic combinations that have a synergistic effect, leading to increased bactericidal activity, higher biofilm penetration and a reduced incidence of antibiotic resistance during the treatment of *S.* *aureus* infection^10,11^. Nevertheless, a recent meta-analysis concluded that the combination antibiotic therapies that have been assessed in patients with MSSA not only failed to reduce mortality, but actually increased the risk of adverse events in humans^12^.

The combination of cloxacillin and fosfomycin is an appealing option for the treatment of MSSA bacteremia. Fosfomycin inhibits the synthesis of *N*-acetylmuramic acid, a precursor of bacterial wall peptidoglycan, and is highly bactericidal against *S.* *aureus*^13^. Interestingly, the addition of fosfomycin to cloxacillin and several other beta-lactam combinations have been shown to have a synergistic effect in vitro, in animal models and in small-scale clinical observational studies^14,15^. To date, however, the use of adjunctive fosfomycin for the treatment of MSSA bacteremia has not been evaluated in a randomized clinical trial.

We conducted an open-label, multicenter, phase III–IV superiority randomized clinical trial (the SAFO trial) to assess whether cloxacillin plus fosfomycin administered for the initial 7 days of therapy achieves better treatment success than cloxacillin alone in hospitalized patients with MSSA bacteremia.

## Results

**Fig. 1:**
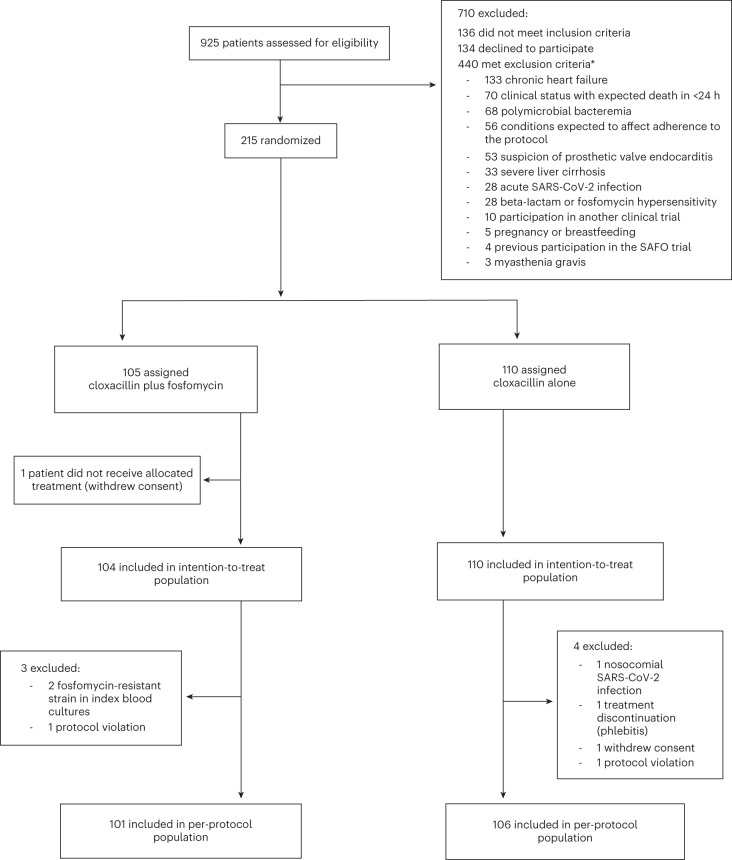
Trial profile. CONSORT diagram indicating participant numbers and disposition throughout the course of the trail. *51 patients had more than one exclusion criterion.

Between 31 May 2019 and 24 February 2022, we assessed 925 patients with MSSA bacteremia for eligibility. After excluding 710 patients who were considered ineligible, we enrolled 215 patients, who were randomly assigned to receive cloxacillin plus fosfomycin (*n* = 105; 49%) or cloxacillin alone (*n* = 110; 51%). One patient assigned to receive cloxacillin plus fosfomycin was excluded before receiving any antibiotic dose owing to withdrawal of consent. Therefore, the primary endpoint was analyzed with the intention-to-treat approach in 214 patients who received at least 1 day of treatment. The analysis of the per-protocol population included 207 patients. The trial profile is shown in Fig. 1. Patients received 2 g of intravenous cloxacillin every 4 h plus 3 g of intravenous fosfomycin every 6 h, or 2 g of intravenous cloxacillin alone every 4 h for the initial 7 days of treatment. Thereafter, the choice and duration of antibiotic therapy was determined by the attending physicians.

The primary endpoint was treatment success at day 7 after randomization, a composite endpoint comprising the following criteria: patient alive, stable or with improved quick sequential organ failure assessment (qSOFA) score, afebrile and with negative blood cultures for MSSA. In a planned interim analysis performed when half of the sample size had been recruited, an independent committee blinded to treatment allocation recommended stopping randomization because its members estimated that it was highly unlikely that statistically significant superiority of the combination therapy would be achieved with full enrollment (data regarding this decision are provided in the Methods).

### Patient characteristics

The patients’ baseline characteristics were similar in the two treatment groups (Table 1). Median age, the proportion of male patients, mean Charlson comorbidity index score and the prevalence of implants were slightly higher in patients receiving cloxacillin alone, and the qSOFA score and the Pitt bacteremia score were similar in the two groups. The main sources of bacteremia at the time of index blood cultures were intravascular catheter, bone and joint infection, and skin and soft tissue infection. Most patients had received an anti-staphylococcal antibiotic in the 72 h preceding randomization. Echocardiography was performed in 77 (74%) of 104 patients receiving cloxacillin plus fosfomycin and in 83 (75%) of 110 patients receiving cloxacillin alone. No significant differences in the percentage of patients undergoing transthoracic (65% versus 75%; relative risk (RR) = 0.87; 95% CI, 0.73–1.03) and transesophageal (15% versus 22%; RR = 0.70; 95% CI, 0.40–1.25) echocardiography were found between treatment groups. A final diagnosis of left-side endocarditis was established at test of cure (TOC) in 4 patients (4%) receiving cloxacillin plus fosfomycin and 11 patients (10%) receiving cloxacillin alone (RR = 0.38; 95% CI, 0.13–1.17). None of the eight patients with prosthetic valves was subsequently diagnosed with prosthetic valve endocarditis. Source of infection control procedures, mainly intravenous catheter removal, were carried out in 57 (55%) of 104 patients receiving cloxacillin plus fosfomycin and 51 (46%) of 110 patients receiving cloxacillin alone (*P* = 0.272). No patient received an additional MSSA-active antibiotic within 7 days after randomization.

**Table 1:** Baseline characteristics in the intention-to-treat population

	Cloxacillin plus fosfomycin (*n* = 104)	Cloxacillin alone (*n* = 110)
Sex		
Male	69 (66%)	81 (74%)
Female	35 (34%)	29 (26%)
Age, median (IQR), years	64 (55–72)	68 (54–77)
Acquisition		
Community-acquired	42 (40%)	36 (33%)
Nosocomial infection	36 (35%)	48 (44%)
Healthcare-associated	26 (25%)	26 (24%)
Time from index blood culture to randomization, median (IQR), days	2 (1–3)	2 (1–3)
Charlson comorbidity index score^a^		
Mean (SD)	4.0 (3.1)	4.7 (3.5)
Score of ≥4	57 (55%)	68 (62%)
qSOFA score^b^		
Mean (SD)	0.3 (0.6)	0.3 (0.6)
Score of ≥1	26 (25%)	23 (21%)
Pitt bacteremia score^c^		
Mean (SD)	0.6 (0.9)	0.5 (0.9)
Score of ≥1	43 (41%)	33 (30%)
Implants	20 (19%)	31 (28%)
Orthopedic	14	16
Pacemaker or indwelling prosthetic valve	2	6
Other intravascular foreign material	4	9
Source of infection at time of index blood culture		
Intravascular catheter	32 (31%)	36 (33%)
Bone and joint	21 (20%)	11 (10%)
Skin and soft tissue	12 (11.5%)	15 (14%)
Not established	14 (13%)	19 (17%)
Urinary	5 (5%)	8 (7%)
Endocarditis	3 (3%)	2 (2%)
Surgical site	6 (6%)	6 (5%)
Pneumonia	2 (2%)	2 (2%)
Other	9 (9%)	11 (10%)
Any anti-staphylococcal antibiotic in the 72 h preceding randomization	99 (95%)	106 (96%)

^a^The Charlson comorbidity index score provides a 10-year mortality risk based on weighted comorbid conditions, ranging from 0 (no comorbid conditions) to 29, a score of 4 being associated with an estimated 10-year survival of 53%.

^b^The qSOFA score identifies patients with suspected infection who are at greater risk of a poor outcome. It uses three criteria, assigning one point for low blood pressure (systolic blood pressure ≤ 100 mmHg), high respiratory rate (≥22 breaths per min) or altered mentation (Glasgow coma score < 15). The score ranges from 0 to 3 points. The presence of 2 or more qSOFA points near the onset of infection was associated with a greater risk of death or prolonged intensive care unit stay.

^c^The Pitt bacteremia score provides a measure of in-hospital mortality risk in patients with bacteremia based on clinical variables. It ranges from 0 to 14 points, with a score of ≥4 being used as an indicator of critical illness and increased risk of death.

### Primary and secondary endpoints

**Fig. 2:**
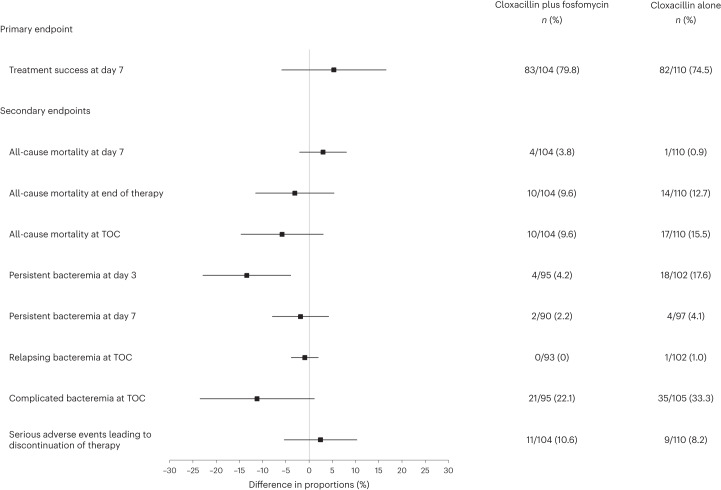
Forest plot of the primary and secondary endpoints in the intention-to-treat population. Data are presented in the plot as absolute difference (percentage in the cloxacillin plus fosfomycin group minus percentage in the cloxacillin alone group) and 95% CIs. Columns on the right show the number of individuals who experienced the event relative to the total number of individuals and the percentage in both groups.

The results for primary and secondary endpoints in the intention-to-treat population are shown in Fig. 2. Table 2 shows primary and secondary endpoints in the intention-to-treat and per-protocol population. In the intention-to-treat population, treatment success at day 7 after randomization was achieved in 83 (79.8%) of 104 patients receiving cloxacillin plus fosfomycin versus 82 (74.5%) of 110 patients receiving cloxacillin alone (risk difference 5.3%; 95% CI, −5.95–16.48; *P* = 0.36). As no statistically significant differences were found in the primary endpoint at day 7, a hierarchical analysis of treatment success at TOC was not performed.

**Table 2:** Primary and secondary endpoints in the intention-to-treat and per-protocol populations

Intention-to-treat population	Cloxacillin plus fosfomycin (*n* = 104)	Cloxacillin alone (*n* = 110)	Risk difference % (95% CI)	*P* value*
Primary endpoint				
Treatment success at day 7	83 (79.8%)	82 (74.5%)	5.3 (−5.95–16.48)	0.360
Secondary endpoints				
All-cause mortality at day 7	4 (3.8%)	1 (0.9%)	2.9 (−2.1–7.97)	0.333
All-cause mortality at end of therapy^a^	10 (9.6%)	14 (12.7%)	−3.1 (−11.53–5.31)	0.453
All-cause mortality at TOC^b^	10 (9.6%)	17 (15.5%)	−5.9 (−14.66–2.98)	0.196
Persistent bacteremia at day 3^c^	4/95 (4.2%)	18/102 (17.6%)	−13.4 (−22.88–−3.99)	0.006
Persistent bacteremia at day 7^d^	2/90 (2.2%)	4/97 (4.1%)	−1.9 (−7.97–4.16)	0.748
Microbiological treatment failure at 14 days^e^	0 (%)	0 (%)	–	–
Relapsing bacteremia at TOC^f^	0/93 (0%)	1/102 (1%)	−0.9 (−3.87–1.91)	1
Complicated bacteremia at TOC^g^	21/95 (22.1%)	35/105 (33.3%)	−11.2 (−23.51–1.06)	0.077
Emergence of fosfomycin-resistant strains at TOC	0 (0%)	0 (0%)	–	–
Length of intensive care unit stay, median (IQR), days	8.0 (3.0–17.0)	4.0 (3.25–8.50)	–	0.355
Duration of intravenous antibiotic treatment, median (IQR), days	14.0 (11.0–22.0)	15.5 (11.0–26.0)	–	0.245
Serious adverse events leading to discontinuation of therapy^h^	11 (10.6%)	9 (8.2%)	2.40 (−5.43–10.22)	0.547

Per-protocol population	Cloxacillin plus fosfomycin (*n* = 101)	Cloxacillin alone (*n* = 106)	Risk difference % (95% CI)	*P* value*
Primary endpoint				
Treatment success at day 7	81 (80.2%)	81 (76.4%)	3.8 (−7.43–15)	0.51
Secondary endpoints				
All-cause mortality at day 7	2 (2%)	0 (0%)	2 (−1.7–5.66)	0.145
All-cause mortality at end of therapy^a^	10 (9.9%)	11 (10.4%)	−0.5 (−8.7–7.75)	0.91
All-cause mortality at TOC^b^	10 (9.9%)	14 (13.2%)	−3.3 (−11.99–5.38)	0.458
Persistent bacteremia at day 3^c^	4/94 (4.3%)	17/99 (17.2%)	−12.9 (−22.43–−3.4)	0.005
Persistent bacteremia at day 7^d^	2/88 (2.3%)	4/95 (4.2%)	−1.9 (−8.13–4.26)	0.684
Microbiological treatment failure at 14 days^e^	0 (%)	0 (%)	–	–
Relapsing bacteremia at TOC^f^	0/91 (0%)	1/99 (1%)	−1 (−3.99–1.97)	1
Complicated bacteremia at TOC^g^	20/93 (21.5%)	34/102 (33.3%)	−11.8 (−24.21–0.56)	0.078
Emergence of fosfomycin-resistant strains at TOC	0 (0%)	0 (0%)	–	–
Length of intensive care unit stay, median (IQR), days	9.0 (4.75–15.8)	4.0 (3.25–8.50)	–	0.168
Duration of intravenous antibiotic treatment, median (IQR), days	14.0 (11.0–22.0)	16.0 (11.0–26.0)	–	0.181
Serious adverse events leading to discontinuation of therapy^h^	10 (9.9%)	6 (5.7%)	4.2 (−4.03–12.51)	0.304

*The *P* values were obtained from a two-sided test for differences in proportions.

^a^End of therapy visit 48 h after the last dose of antibiotic treatment.

^b^TOC visit 12 weeks after randomization.

^c^At least one positive blood culture for MSSA at day 3.

^d^At least one positive blood culture for MSSA at day 7.

^e^Defined as a positive sterile site culture for MSSA at least 14 days after randomization.

^f^At least one positive blood culture for MSSA at least 72 h after a preceding negative culture at TOC.

^g^Defined as persistent bacteremia, endocarditis, metastatic emboli or the presence of prosthetic devices at TOC.

^h^During the first 7 days after randomization.

In an exploratory analysis, there were no significant differences in the primary endpoint between patients receiving cloxacillin plus fosfomycin and those receiving cloxacillin alone, excluding 68 patients with catheter-related bacteremia (57 (79.2%) of 72 versus 55 (74.3%) of 74; risk difference 4.9%; 95% CI, −8.83–18.52; *P* = 0.48) and analyzing exclusively 66 patients who had high-risk bacteremia (17 (70.8%) of 24 versus 33 (75%) of 44; risk difference 4.2%; 95% CI, −18.07–26.4; *P* = 0.71).

**Fig. 3:**
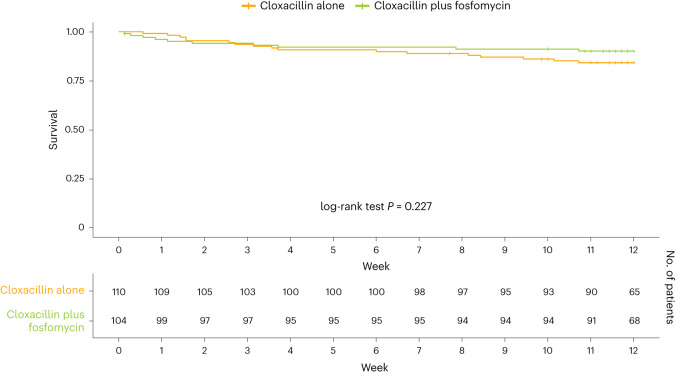
Kaplan–Meier survival estimates of all-cause mortality during follow-up. Survival curves for all-cause mortality are plotted for cloxacillin plus fosfomycin and cloxacillin alone. The log-rank test was used to compare both survival curves.

Also, there were no significant differences in secondary outcomes, including all-cause mortality, at day 7, end of therapy and TOC visits, persistent bacteremia at day 7 after randomization, relapsing bacteremia at TOC, complicated bacteremia, duration of intravenous antibiotic treatment, and serious adverse events leading to discontinuation of therapy during the first 7 days after randomization (Extended Data Table 1). No emergence of fosfomycin-resistant MSSA strains was observed during follow-up. The only significant difference in secondary outcomes was observed in persistent bacteremia at day 3 after randomization, which occurred in 4 (4.2%) of 95 patients receiving cloxacillin plus fosfomycin and in 18 (17.6%) of 102 patients receiving cloxacillin alone (risk difference −13.4%; 95% CI, −22.88–−3.99; *P* = 0.006). Figure 3 shows the Kaplan–Meier survival estimates of all-cause mortality in both treatment groups during follow-up (log-rank test, *P* = 0.227). Per-protocol analyses of primary and secondary endpoints produced similar results to those of the intention-to-treat population (Table 2).

As shown in Table 2, the duration of intravenous antibiotic therapy was similar in the two treatment groups. Overall, the median duration of total antibiotic therapy was 23.5 days (interquartile range (IQR) 14.0–42.0) in patients receiving cloxacillin and fosfomycin and 28.0 days (IQR 15.0–45.8) in those receiving cloxacillin alone. The median duration of fosfomycin therapy was 8 days (IQR 8.0–8.0). We performed a pharmacokinetic analysis in a subgroup of seven patients treated with cloxacillin plus fosfomycin and seven patients treated with cloxacillin alone. A total of 23 cloxacillin pre-dose (minimum concentration (*C*_min_)) samples, 22 cloxacillin post-dose (maximum concentration (*C*_max_)) samples, 9 fosfomycin *C*_min_ samples and 7 fosfomycin *C*_max_ samples were collected. Median cloxacillin *C*_min_ and *C*_max_ were 62.20 mg l^−^^1^ (IQR 22–88) and 89.91 mg l^−1^ (IQR 51.4–129.9), respectively. Median fosfomycin *C*_min_ and *C*_max_ were 99.50 mg l^−1^ (IQR 87–121.2) and 301.40 mg l^−1^ (IQR 173.5–382), respectively.

Table 3 shows adverse events in the intention-to-treat population. The number of serious adverse events at TOC was similar in the two treatment groups; 42 (40%) of 104 patients receiving cloxacillin and fosfomycin and 48 (44%) of 110 patients treated with cloxacillin alone. The most frequent adverse events were hypokalemia, hypocalcemia, acute heart failure and gastrointestinal disorders. The only significant difference was observed in the case of hypocalcemia. Serious adverse events occurred at a median of 13 days (IQR 3.0–43.5) after fosfomycin initiation. A description of all adverse events according to system organ class reported in both treatment groups is provided in Extended Data Table 2.

**Table 3:** Adverse events in the intention-to-treat population

	Cloxacillin plus fosfomycin (*n* = 104)	Cloxacillin alone (*n* = 110)	Risk difference % (95% CI)	*P* value*
Any serious adverse event at TOC	42 (40.4%)	48 (43.6%)	−3.22 (−17.41–10.91)	0.732
Main adverse events at TOC^a^				
Hypokalemia (<3 mmol L^−1^)	18 (17.31%)	11 (10%)	7.31 (−2.81–17.42)	0.173
Hypocalcemia (<2.0 mmol L^−1^)	15 (14.42%)	5 (4.55%)	9.92 (1.15–18.61)	0.018
Acute heart failure	6 (5.77%)	6 (5.45%)	0.27 (−6.17–6.8)	1.000
Gastrointestinal disorders	7 (6.73%)	6 (5.45%)	1.23 (−7.58–6.39)	0.917

*The *P* values were obtained from a two-sided test for differences in proportions.

^a^Adverse events occurring in >4 patients.

## Discussion

This open-label, phase III–IV superiority randomized clinical trial conducted in 19 Spanish hospitals aimed to evaluate whether the combination of cloxacillin and fosfomycin achieved better treatment success than cloxacillin alone in patients with MSSA bacteremia. The primary endpoint was chosen based on the recommendations of international experts that proposed primary endpoints for use in clinical trials comparing treatment options for bloodstream infections in adults^16^. We chose day 7 for the primary endpoint as it seemed an appropriate timepoint to evaluate the effect of antibiotic treatment on the initial response and the early resolution of the infection.

The main finding of our trial is that cloxacillin plus fosfomycin did not achieve better treatment success at day 7 than cloxacillin alone among patients with MSSA bacteremia. Secondary endpoints, including adverse events leading to discontinuation of therapy, were similar in the two treatment groups, with the exception of persistent bacteremia at day 3, which was less common in the combination treatment arm.

The results of our study are in line with the findings of the few randomized clinical trials carried out to date assessing different antibiotic combinations, which have also failed to improve treatment success rates and outcomes in patients with MSSA bacteremia and endocarditis, as shown in a recent meta-analysis^12^. A multicenter, randomized, double-blind, placebo-controlled trial (the ARREST trial)^17^ conducted in 29 hospitals in the United Kingdom evaluated whether adjunctive rifampicin improved the outcomes of adult patients with *S.* *aureus* bacteremia, of whom 6% had infection with methicillin-resistant strains. In that trial, adjunctive rifampicin provided no overall benefit over standard antibiotic therapy in terms of avoiding treatment failure, disease recurrence, or death at 12 weeks after randomization. Moreover, a recent randomized controlled trial performed at two hospitals in Canada evaluating the efficacy of adjunctive daptomycin given with either cloxacillin or cefazolin for the treatment of MSSA bacteremia found that it did not shorten the duration of bacteremia (the primary endpoint) and did not improve 90-day mortality^18^.

As stated above, we found that persistent bacteremia at day 3 after randomization was less frequent in patients receiving cloxacillin plus fosfomycin than in patients receiving cloxacillin alone. However, this finding did not translate into improved survival at day 7. This result contrasts with those of some observational studies that have found that each day of persistent bacteremia is associated with increased mortality^6,7^. Overall, the lack of improvement in survival despite the reduction in persistent bacteremia at day 3 suggests that other factors may be at play. Indeed, persistent bacteremia could be a surrogate marker of a high-risk source of infection, and reducing the number of days with bacteremia may not be enough to outweigh other complications^19^. Further investigation is needed to fully understand the relationship between persistent bacteremia and mortality.

We did not find significant differences in all-cause mortality at day 7 or at end of therapy and TOC visits. Nevertheless, mortality at TOC was higher in patients treated with cloxacillin alone, although the difference was not statistically significant. Of note, median age, the proportion of male patients, mean Charlson comorbidity index score and the prevalence of implants were slightly higher in patients receiving cloxacillin alone, who were also more likely to have a high-risk source of bacteremia, including endocarditis at TOC. Moreover, mortality was low in both treatment groups, and the trial was not powered to detect survival differences.

We found similar rates of adverse events leading to treatment discontinuation during the first 7 days of therapy in the two study groups. In a previous trial comparing daptomycin plus fosfomycin versus daptomycin alone in patients with methicillin-resistant *S.* *aureus* bacteremia, adverse events were more frequent among those receiving fosfomycin^20^. However, in that study, the duration of fosfomycin therapy ranged from 2 to 6 weeks, considerably longer than in the present trial, in which it was 8 days. In the current study, fosfomycin was administered over a 4-hour period as suggested elsewhere^21^, and attending physicians at the participating hospitals were advised to use supplementary potassium and furosemide to avoid sodium overload in patients receiving cloxacillin plus fosfomycin. The fosfomycin dose used in this trial was chosen according to pharmacokinetic data reported in previous studies^13,22^. Interestingly, in our pharmacokinetic study conducted in a small subgroup of patients, high pre-dose C_min_ and post-dose C_max_ of fosfomycin were achieved.

Our study has limitations. The first is the open-label design, which may have introduced a bias in the assessment of treatment success. Nevertheless, this limitation was mitigated by including objective data in the composite primary endpoint, which was also adjudicated by an independent committee blinded to treatment allocation. It should be noted that our trial mainly focused on treatment effect during the first 7 days (when fosfomycin was administered in the combination treatment arm) and assessed relevant secondary endpoints at TOC (12 weeks after randomization). Therefore, we cannot rule out disease recurrence or relapse occurring beyond 12 weeks after randomization. Unfortunately, there are no standardized primary endpoints to be used in trials comparing different strategies for antibiotic treatment of MSSA bacteremia, and efforts should be made to reach consensus regarding the endpoints that should be used in future trials. Another limitation of our study is that it was conducted in a single country, and its findings might not be generalizable to other populations. Furthermore, when enrollment of half of the sample size had been achieved, the independent committee raised no concerns regarding safety, but mentioned the differences between the success rate specified in the sample size calculation and the rate observed in the planned interim analysis, and recommended ceasing patient recruitment owing to futility. Moreover, the number of patients who had high-risk MSSA bacteremia was relatively low, and the trial was not powered to detect survival differences. Finally, our trial did not include patients with prosthetic endocarditis, therefore we cannot draw conclusions about the hypothetical benefits of adjunctive fosfomycin in this setting.

In conclusion, cloxacillin plus fosfomycin did not achieve better treatment success at day 7 of therapy than cloxacillin alone in hospitalized adult patients with MSSA bacteremia. Further large randomized controlled trials should be conducted to evaluate new strategies of treatment aimed at improving outcomes in patients with MSSA bacteremia. Ideally, these trials should be designed taking into account the intrinsic heterogeneity of the infection, by using a more stratified and personalized approach and by including a long-term follow-up.

## Methods

### Study design and setting

We performed an open-label, phase III–IV superiority randomized clinical trial of patients with MSSA bacteremia at 19 Spanish university hospitals (the SAFO trial). Participants were recruited from May 2019 to February 2022. Before inclusion in the trial, all patients or legal representatives provided written informed consent. All participants were able to withdraw from the study at any time without further explanation. The study was authorized by the Spanish Medicines and Healthcare Products Regulatory Agency (AEMPS; 18-0905) and by the Bellvitge University Hospital Ethics Committee (AC069/18). The protocol has been published elsewhere^23^ and followed the Standard Protocol Items: Recommendations for Interventional Trials (SPIRIT) initiative^24^. The trial was conducted in agreement with the principles of the Declaration of Helsinki, the Good Clinical Practice guidelines and the current local legislation. The patients’ personal and clinical information was managed in accordance with European regulation (2016/679) and Spanish legislation. The results are presented following the Consolidated Standards of Reporting Trials (CONSORT) statement^25^. The trial is registered in the EudraCT (2018-001207-37) and ClinicalTrials.gov (NCT03959345) databases.

### Participants

Adult patients aged ≥18 years with at least one blood culture positive for MSSA ≤ 72 h before randomization, with evidence of active infection, were considered eligible for inclusion in the study. Treatment with any anti-staphylococcal antibiotic ≤72 h preceding randomization was allowed. Exclusion criteria were severe clinical status with expected death in <24 h; severe liver cirrhosis (Child–Pugh C); moderate-to-severe chronic heart failure (New York Heart Association functional classification, class III–IV); suspicion of prosthetic valve endocarditis; history of significant allergy to beta-lactam antibiotics or fosfomycin (defined as previous type 1 hypersensitivity reaction to any beta-lactam antibiotics or fosfomycin, or history of serious non-type 1 hypersensitivity reaction to any penicillin or fosfomycin); known non-susceptibility of *S.* *aureus* to fosfomycin; polymicrobial bacteremia; pregnancy or breastfeeding at the time of inclusion; myasthenia gravis; participation in another clinical trial; previous participation in the present clinical trial; and social problems or cognitive or psychiatric impairment that might be expected to affect adherence to the study. Acute SARS-CoV-2 infection was added as an exclusion criterion by a protocol amendment after the start of the pandemic. This amendment was approved by the Hospital Universitari de Bellvitge Ethics Committee and by the AEMPS on 29 November 2020. The source of MSSA bacteremia was determined following criteria published elsewhere^26^. Accordingly, nosocomial MSSA bacteremia was defined as a positive blood culture obtained from patients who had been hospitalized for 48 h or longer. Healthcare-associated bacteremia was defined as a positive MSSA blood culture obtained from a patient at the time of hospital admission or within 48 h of admission if the patient fulfilled any of the following criteria: received intravenous therapy at home or specialized home care in the 30 days before bacteremia; attended a hospital or hemodialysis clinic, or received intravenous chemotherapy in the 30 days before bacteremia; was hospitalized in an acute care hospital for two or more days in the 90 days before bacteremia; resided in a nursing home or long-term care facility. Community-acquired MSSA bacteremia was defined as a positive blood culture obtained at the time of hospital admission for patients who did not fit the criteria for a healthcare-associated infection.

### Randomization and masking

Participants were randomly assigned (1:1) to receive cloxacillin plus fosfomycin or cloxacillin alone, for the initial 7 days of treatment. A centralized electronic computer randomization schedule was developed by the Biostatistics Unit at the Bellvitge Biomedical Research Institute (IDIBELL). The randomization was performed in computer-generated variable blocks ranging from four to six patients stratified per center to conceal the sequence until the intervention was assigned. The code numbers for eligible participants were assigned in ascending sequential order. The allocation list was stored at IDIBELL and was not available to any member of the research team. At each participating hospital, patients who provided written informed consent and met the study criteria were randomized by investigators, who obtained the assigned treatment and code number from a computer-assisted website.

### Procedures

Participants were randomly assigned to receive cloxacillin plus fosfomycin or cloxacillin alone. Cloxacillin sodium (Cloxacillin, Normon) was administered intravenously by a 60-min infusion at a dose of 2 g every 4 h, and fosfomycin sodium (Fosfocin, ERN), was given intravenously by 4-hour infusion every 6 h at a dose of 3 g. The intravenous fosfomycin dose was selected according to pharmacokinetic and/or pharmacodynamic data reported elsewhere^13^. Antibiotic dosage was adjusted according to creatinine clearance^23^. Fosfomycin was administered during the first 7 days of therapy to obtain a synergistic effect and high bactericidal activity, and to avoid serious adverse events based on our previous experience^20^.

The antibiotic regimens were administered during the first 7 days after randomization. Thereafter, the choice of antibiotic therapy was determined by the attending physicians. In general, uncomplicated bacteremia was treated for 10–14 days, and complicated bacteremia (defined as infection with hematogenous seeding, progression of infection beyond the primary focus, persistent bacteremia, skin lesions suggestive of acute systemic infection, presence of non-catheter device, and hemodialysis) for 4–6 weeks at least, depending on the source of the infection and other clinical considerations. Intravenous catheters and other non-catheter devices, such as pacemakers, were removed if they were considered the source of bacteremia. Transthoracic and transesophageal echocardiograms were performed at the discretion of the attending physicians.

Patients were assessed at randomization and at days 3 and 7 by at least one of the researchers, and were followed up daily by an infectious disease specialist. Scheduled visits were performed for all participants at the end of therapy (48 h after the last dose of antibiotic treatment) and at the TOC visit (12 weeks after randomization). TOC visits were performed face-to-face or by telephone in cases with no symptoms of infection. Blood cultures were obtained at days 3 and 7, at the end of therapy and at TOC (if symptoms or signs of infection were present). Moreover, blood cultures, hematological and biochemistry analyses were obtained whenever considered necessary by the attending physicians.

*S.* *aureus* isolates from blood cultures were identified and subjected to antimicrobial susceptibility testing by the microbiology department at each participating hospital. Fosfomycin susceptibility was routinely tested on all *S.* *aureus* isolates. Strains were anonymized and stored at −70 °C until being shipped to the central laboratory at the microbiology department of Hospital Universitari de Bellvitge. Once received, the identification of each isolate was confirmed by MALDI–TOF (matrix-assisted laser desorption/ionization–time of flight) mass spectrometry (MALDI Biotyper, Bruker Daltonics). Antimicrobial susceptibility was determined by microdilution using commercially available panels (MicroScan, Beckman Coulter) and assessed according to the European Committee on Antimicrobial Susceptibility Testing (EUCAST) guidelines^27^.

Total plasma concentrations of cloxacillin and fosfomicyn were measured in a subgroup of patients by a previously validated method based on ultra-high-performance liquid chromatography–tandem mass spectrometry in human plasma^28^.

### Outcomes

The primary study endpoint was treatment success at day 7, a composite endpoint defined as the presence of all of the following criteria: patient alive, stable or with improved qSOFA score compared with baseline, afebrile and with negative blood cultures for MSSA. The primary endpoint was adjudicated by an independent committee blinded to the antibiotic therapy received by participants. Withdrawal of study medication for any reason before day 7 was considered treatment failure. A hierarchical analysis of treatment success had been planned at TOC only if there had been statistical differences in the primary endpoint at day 7. The analysis at day 7 would provide an early indication of whether the antibiotic was effective in controlling the infection.

The secondary clinical endpoints were all-cause mortality at day 7, end of therapy and TOC visits, persistent bacteremia (at least one positive blood culture) at day 3 and day 7 after randomization, microbiological treatment failure (defined as a positive sterile site culture for MSSA at least 14 days after randomization), relapsing bacteremia (defined as at least one positive blood culture for MSSA at least 72 h after a preceding negative culture) assessed at TOC, complicated bacteremia (defined as persistent bacteremia, endocarditis, metastatic emboli or the presence of prosthetic devices), emergence of fosfomycin-resistant strains, length of intensive care unit stay, duration of intravenous antibiotic treatment, and serious adverse events leading to discontinuation of therapy during the first 7 days after randomization.

A systematic, prioritized, risk-based approach to the monitoring of adverse events was applied to ensure that the trial was conducted, recorded and reported in accordance with good clinical practices^29^. Adverse events were recorded in all patients who received at least one dose of the study medication. Clinical laboratory tests, vital signs and other safety assessments were performed at scheduled visits. Serious adverse events (including death) leading to discontinuation of therapy were considered key safety parameters.

All data were recorded on a secure web application for building and managing online databases (REDCap)^30^. The study endpoints were assessed by an independent committee blinded to treatment allocation and to patient identity.

### Statistical analysis

On the basis of our own experience^5^, we expected a level of treatment success of 74% among patients with MSSA bacteremia receiving cloxacillin alone. A sample size of 183 patients per treatment arm was calculated to be able to reject the null hypothesis of equal effect with a power of 80% and a significance level of 5% for a 12% difference in treatment success among patients treated with cloxacillin plus fosfomycin. A dropout rate of 5% was anticipated. On 10 February 2022, the planned interim analysis to evaluate the safety and feasibility of the trial was performed when half of the sample size had been achieved (data from 188 participants). The independent committee, which was blinded to antibiotic treatment allocation and comprised specialists in biostatistics, pharmacology and infectious diseases, raised no concerns regarding safety. However, their interim analysis showed nearly identical treatment success at 7 days in the two treatment groups. The independent committee mentioned the differences between the success rate specified in the sample size calculation (86% for cloxacillin plus fosfomycin and 74% for cloxacillin alone) and the rate observed in the interim analysis (78.8% and 76.6%). Compared to the expected difference of 12% at the end of the trial, a difference of 2.2% was observed at the interim analysis. Given these results, the estimated conditional power was lower than 10%, and the probability of rejecting the null hypothesis was lower than 0.1. This was not considered acceptable because the difference was far from clinical significance. Therefore, the independent committee recommended ceasing patient recruitment because of futility, as it was very unlikely that continuing the study would yield significant differences in the primary endpoint between the two treatment arms. The trial’s steering committee closed trial recruitment on 24 February 2022.

Data for the primary and secondary endpoints were analyzed with the intention-to-treat approach and per protocol. The intention-to-treat analysis included all randomly assigned patients who received at least one day of treatment. As the two analyses produced virtually the same results, only the intention-to-treat analysis is presented in detail. All patients who received at least one dose of treatment were included in the safety analysis. Main efficacy analyses and the proportion of treatment success at day 7 were compared between groups using a two-sided chi-squared test. Relative risks for study outcomes were calculated and reported with 95% confidence intervals. The incidences of events in secondary outcomes were compared using the chi-squared test, Fisher’s exact test or the Mann–Whitney test. Kaplan–Meier curves for survival were constructed and compared using the log-rank test. All analyses and data management were performed with R software, v.4.0.4 or later^31^. The most relevant R packages used were dplyr, REDCapDM, compareGroups and survival^32–34^.

### Reporting summary

Further information on research design is available in the Nature Portfolio Reporting Summary linked to this article.

## Online content

Any methods, additional references, Nature Portfolio reporting summaries, source data, extended data, supplementary information, acknowledgements, peer review information; details of author contributions and competing interests; and statements of data and code availability are available at 10.1038/s41591-023-02569-0.

### Supplementary information

Reporting Summary

## Data Availability

Individual patient data cannot be shared because of privacy restrictions. Raw anonymized data relating to primary and secondary outcomes and safety can be shared upon request. Depending on the data requested, we will need to consult with the institutional review board at Hospital Universitari de Bellvitge. Requests for data can be sent to the corresponding authors (M.P. and J.C.). All requests will be answered within 4 weeks.

## Extended data

**Extended Data Table 1:** Adverse events leading to treatment discontinuation during the first seven days from randomization

	Cloxacillin plus Fosfomycin (n = 104)	Cloxacillin alone (n = 110)
Patients with adverse events leading to treatment discontinuation	11 (10.6%)	9 (8.2%)
Adverse events leading to treatment discontinuation*	13 (11.3%)	10 (10.6%)
Cardiac disorders	1	1
Gastrointestinal disorders	5	1
General disorders and administration site conditions	1	0
Hepatobiliary disorders	1	0
Infections and infestations	0	1
Investigations	2	0
Metabolism and nutrition disorders	3	2
Neoplasms benign, malignant and unspecified	0	1
Renal and urinary disorders	0	2
Respiratory, thoracic and mediastinal disorders	0	1
Skin and subcutaneous tissue disorders	0	1

* Percentage computed with respect to the total number of adverse events (115 for cloxacillin plus fosfomycin and 94 for cloxacillin alone).

**Extended Data Table 2:** Adverse events according to system organ class*

	Cloxacillin plus Fosfomycin (n = 104)	Cloxacillin alone (n = 110)
Blood and lymphatic system disorders	1	0
Cardiac disorders	9	7
Gastrointestinal disorders	7	6
General disorders and administration site conditions	4	7
Hepatobiliary disorders	1	0
Infections and infestations	13	20
Injury, poisoning and procedural complications	3	0
Investigations	1	0
Metabolism and nutrition disorders	25	20
Musculoskeletal and connective tissue disorders	0	2
Neoplasms benign, malignant and unspecified (including cysts and polyps)	5	4
Nervous system disorders	1	1
Product issues	1	0
Renal and urinary disorders	2	3
Respiratory, thoracic and mediastinal disorders	2	6
Surgical and medical procedures	4	1
Vascular disorders	3	4

*Adverse events are reported based on Conventional International Conference on Harmonization definitions. Patients could have more than one adverse event.
