# Supplementary material for: Cloxacillin plus fosfomycin versus cloxacillin alone for methicillin-susceptible Staphylococcus aureus bacteremia: a randomized trial
Source: Nat Med. 2023 Oct 2;29(10):2518–25. doi: 10.1038/s41591-023-02569-0 (PMC10579052; doi:10.1038/s41591-023-02569-0)
Supplement: Supplementary file 1 — Reporting Summary [file 41591_2023_2569_MOESM1_ESM.pdf]

nature portfolio

Corresponding author(s): Miquel Pujol and Jordi Carratalà

Last updated by author(s): Aug 24, 2023

# Reporting Summary

Nature Portfolio wishes to improve the reproducibility of the work that we publish. This form provides structure for consistency and transparency in reporting. For further information on Nature Portfolio policies, see our Editorial Policies and the Editorial Policy Checklist.

## Statistics

For all statistical analyses, confirm that the following items are present in the figure legend, table legend, main text, or Methods section.

| n/a | Confirmed | |
|---|---|---|
| ☐ | ☒ | The exact sample size (*n*) for each experimental group/condition, given as a discrete number and unit of measurement |
| ☐ | ☒ | A statement on whether measurements were taken from distinct samples or whether the same sample was measured repeatedly |
| ☐ | ☒ | The statistical test(s) used AND whether they are one- or two-sided<br>*Only common tests should be described solely by name; describe more complex techniques in the Methods section.* |
| ☐ | ☒ | A description of all covariates tested |
| ☐ | ☒ | A description of any assumptions or corrections, such as tests of normality and adjustment for multiple comparisons |
| ☐ | ☒ | A full description of the statistical parameters including central tendency (e.g. means) or other basic estimates (e.g. regression coefficient) AND variation (e.g. standard deviation) or associated estimates of uncertainty (e.g. confidence intervals) |
| ☐ | ☒ | For null hypothesis testing, the test statistic (e.g. *F*, *t*, *r*) with confidence intervals, effect sizes, degrees of freedom and *P* value noted<br>*Give P values as exact values whenever suitable.* |
| ☒ | ☐ | For Bayesian analysis, information on the choice of priors and Markov chain Monte Carlo settings |
| ☒ | ☐ | For hierarchical and complex designs, identification of the appropriate level for tests and full reporting of outcomes |
| ☐ | ☒ | Estimates of effect sizes (e.g. Cohen's *d*, Pearson's *r*), indicating how they were calculated |

*Our web collection on statistics for biologists contains articles on many of the points above.*

## Software and code

Policy information about availability of computer code

| | |
|---|---|
| Data collection | REDCap v9.0 |
| Data analysis | R software v4.0.5 (March 31, 2021) |

For manuscripts utilizing custom algorithms or software that are central to the research but not yet described in published literature, software must be made available to editors and reviewers. We strongly encourage code deposition in a community repository (e.g. GitHub). See the Nature Portfolio guidelines for submitting code & software for further information.

## Data

Policy information about availability of data

All manuscripts must include a data availability statement. This statement should provide the following information, where applicable:

- Accession codes, unique identifiers, or web links for publicly available datasets
- A description of any restrictions on data availability
- For clinical datasets or third party data, please ensure that the statement adheres to our policy

The manuscript includes a data availability statement at the end of the methods section.

## Human research participants

Policy information about studies involving human research participants and Sex and Gender in Research.

| | |
|---|---|
| Reporting on sex and gender | Sex; male or female |
| Population characteristics | Age, sex, site of acquisition of MSSA bacteremia, Charlson comorbidity score, qSOFA, Pitt bacteremia score, implants, source of infection at time of index blood cultures, previous anti-staphylococcal antibiotic in the previous 72 hours. Table 1 of the manuscript. |
| Recruitment | Participants were recruited from May 2019 to February 2022. Before inclusion in the trial, all patients or legal representatives provided written informed consent. All participants were able to withdraw from the study at any timepoint without further explanation. Adult patients aged ≥ 18 years with at least one blood culture positive for MSSA ≤72 hours before randomization, with evidence of active infection, were considered eligible for inclusion in the study. Material and Methods |
| Ethics oversight | The study was authorized by the Spanish Medicines and Healthcare Products Regulatory Agency (AEMPS; 18-0905) and by the Bellvitge University Hospital Ethics Committee (AC069/18). |

Note that full information on the approval of the study protocol must also be provided in the manuscript.

# Field-specific reporting

Please select the one below that is the best fit for your research. If you are not sure, read the appropriate sections before making your selection.

☒ Life sciences ☐ Behavioural & social sciences ☐ Ecological, evolutionary & environmental sciences

For a reference copy of the document with all sections, see nature.com/documents/nr-reporting-summary-flat.pdf

# Life sciences study design

All studies must disclose on these points even when the disclosure is negative.

| | |
|---|---|
| Sample size | On the basis of our own experience5, we expected a level of treatment success of 74% among patients with MSSA bacteremia receiving cloxacillin alone. A sample size of 183 patients per treatment arm was calculated in order to be able to reject the null hypothesis of equal effect with a power of 80% and a significance level of 5% for a 12% difference in treatment success among patients treated with cloxacillin plus fosfomycin. A dropout rate of 5% was anticipated. |
| Data exclusions | No data were excluded from the analyses |
| Replication | Randomized controlled trial. Primary and secondary endpoints well defined and assessed by an independent committeee blinded to antibiotic treatment allocation. |
| Randomization | Participants were randomly assigned (1:1) to receive cloxacillin plus fosfomycin or cloxacillin alone, for the initial seven days of treatment. A centralized electronic computer randomization schedule was developed by the Biostatistics Unit at the Bellvitge Biomedical Research Institute (IDIBELL). The randomization was performed in computed-generated variable blocks ranging from four to six patients stratified per center, so as to conceal the sequence until the intervention was assigned. The code numbers for eligible participants were assigned in ascending sequential order. The allocation list was stored at IDIBELL and was not available to any member of the research team. At each participating hospital, patients who provided written informed consent and met the study criteria were randomized by investigators, who obtained the assigned treatment and code number from a computer-assisted website. |
| Blinding | The investigators of each participant center were not blinded to group allocation but endpoints were assessed by an independent committee blinded to antibiotic treatment allocation. |

# Reporting for specific materials, systems and methods

We require information from authors about some types of materials, experimental systems and methods used in many studies. Here, indicate whether each material, system or method listed is relevant to your study. If you are not sure if a list item applies to your research, read the appropriate section before selecting a response.

## Materials & experimental systems

| n/a | Involved in the study |
|---|---|
| ☒ | ☐ Antibodies |
| ☒ | ☐ Eukaryotic cell lines |
| ☒ | ☐ Palaeontology and archaeology |
| ☒ | ☐ Animals and other organisms |
| ☐ | ☒ Clinical data |
| ☒ | ☐ Dual use research of concern |

## Methods

| n/a | Involved in the study |
|---|---|
| ☒ | ☐ ChIP-seq |
| ☒ | ☐ Flow cytometry |
| ☒ | ☐ MRI-based neuroimaging |

# Clinical data

Policy information about clinical studies

All manuscripts should comply with the ICMJE guidelines for publication of clinical research and a completed CONSORT checklist must be included with all submissions.

Clinical trial registration: The trial is registered in the EudraCT (2018-001207-37) and ClinicalTrials.gov (NCT03959345) databases.

Study protocol: The study protocol was published in BMJ Open 2021; 11: e051208 and is provided in the supplementary material

Data collection: Participants were recruited from May 2019 to February 2022 in 19 Spanish hospitals.

Outcomes: The primary study endpoint was treatment success at day 7, a composite endpoint defined when all the following criteria were met after randomization: patient alive at day 7, stable or improved quick-Sequential Organ Failure Assessment (qSOFA) score compared with baseline at day 7 and fever resolved at day 7 and negative blood cultures for MSSA at day 7, assessed by an independent committee blinded to the antibiotic therapy received by participants. Withdrawal of study medication for any reason before day 7 was considered treatment failure. A hierarchical analysis of treatment success had been planned at TOC only if there had been statistical differences in the primary endpoint at day 7.
The secondary clinical endpoints were all-cause mortality at day 7, EOT and TOC visits, persistent bacteremia (at least one positive blood culture) at day 3 and persistent bacteremia at day 7 after randomization, microbiological failure at 14 days after randomization, relapsing bacteremia (defined as at least one positive blood culture for MSSA at least 72 hours after a preceding negative culture) assessed at TOC, complicated bacteremia (defined as persistent bacteremia, endocarditis, metastatic emboli or the presence of prosthetic devices), emergence of fosfomycin-resistant strains, lenght of intensive care unit stay, duration of intravenous antibiotic treatment, and serious adverse events leading to discontinuation of therapy during the first seven days after randomization.
A systematic, prioritized, risk-based approach to the monitoring of adverse events was applied to ensure that the trial was conducted, recorded, and reported according to good clinical practices28. Adverse events were recorded in all patients who received at least one dose of the study medication. Clinical laboratory tests, vital signs, and other safety assessments were performed at scheduled visits. Mortality and serious adverse events leading to discontinuation of therapy were considered key safety parameters. All data were recorded on a secure web application for building and managing online databases (REDCap)29. The study endpoints were assessed by an independent committee blinded to treatment allocation and to patient identification.
